# Overcoming Barriers to *Walk With Ease* Implementation in Community Organizations

**DOI:** 10.1177/15248399211002851

**Published:** 2021-04-02

**Authors:** Leigha H. Vilen, Mary Altpeter, Leigh F. Callahan

**Affiliations:** 1 University of North Carolina at Chapel Hill, Chapel Hill, NC, USA; 2 University of Wisconsin, Madison, WI, USA

**Keywords:** arthritis, physical activity/exercise, program planning and evaluation, community organizations

## Abstract

**Introduction:**

The Arthritis Foundation’s Walk With Ease (WWE) program has been shown to reduce arthritis symptoms and increase physical performance for up to 1 year. However, research on community-based WWE implementation is limited. The purpose of this study was to examine early implementation at community organizations that received 1-year WWE implementation grants from the Osteoarthritis Action Alliance.

**Method:**

Program managers at five Osteoarthritis Action Alliance grantee organizations participated in 45-minute telephone interviews. Interviewees represented organizations with the highest WWE enrollment at 6 months (*n* = 3, >30% of goal enrollment) and no enrollment at 6 months (*n* = 2). The Planning for Sustainability framework guided qualitative analysis of factors affecting early implementation.

**Results:**

All grantees were confident in WWE’s evidence base, thought it a beneficial supplement to other programming, stressed the importance of senior leadership support for WWE, and engaged community partners for marketing support and as walking sites. Implementation experiences unique to low enrollment grantees were (1) recent major structural changes within their organization, (2) difficulties in communicating logistics internally, and (3) difficulties in balancing WWE with other responsibilities. All organizations experienced barriers that required altering their original implementation plans; however, practical solutions like adapting the program to improve flexibility, training organizational staff as leaders, and utilizing community partnerships served to address multiple barriers simultaneously.

**Discussion:**

Building organizational capacity by overcoming early barriers is a key element of early implementation. Our findings offer concrete solutions to early WWE implementation barriers and suggest the need for further research on adaptations to improve WWE’s flexibility in community organizations.

Doctor-diagnosed arthritis affects 54.4 million (22.7%) adults and is the number one cause of disability in the United States (Barbour et al., 2017; Centers for Disease Control and Prevention [CDC], 2009). Regular physical activity improves pain and physical function by about 40% in all forms of arthritis (Kelley et al., 2011). Despite the benefits, fewer adults with arthritis meet CDC physical activity guidelines compared with the general population (CDC, 2011; Wallis et al., 2013).

The Arthritis Foundation’s Walk With Ease (WWE) program, an evidence-based physical activity intervention, can reduce arthritis symptoms and increase physical performance for up to 1 year (Callahan et al., 2011). It has similar benefits with diverse racial/ethnic groups and in community-based locations like workplaces (Callahan et al., 2016; Nyrop et al., 2011; Wyatt et al., 2014). It was also feasible and efficacious when scaled up for widespread implementation at 28 community sites in Oregon (Conte et al., 2016). WWE’s evidence base is strong; however, research on its implementation in community-based settings is limited (Conte et al., 2019; Conte et al., 2017). This article evaluates early implementation of WWE in five community-based organizations (CBOs) that received 1-year grants for program delivery. We examine organizational characteristics and implementation activities during the first 6 months of their implementation grants and identify key differences between organizations that were successful with implementation and those that struggled. We also describe implementation barriers and provide practical strategies that organizations employed to overcome them.

## Study Context: The Osteoarthritis Action Alliance WWE Expansion Small Grants

Proceeding through the early stages of program implementation requires significant resource investment, as organizations build capacity and develop routinized procedures to sustain a program over time (Fixsen et al., 2005). In 2016, recognizing the importance of studying and supporting community-based program implementation, the CDC awarded the Osteoarthritis Action Alliance (OAAA) a 5-year grant to identify and implement effective dissemination and delivery models for WWE. In April 2017, through a competitive application process, the OAAA awarded 1-year small grants to 15 CBOs to enroll at least 150 participants in WWE. WWE was chosen because it is low cost and available in both instructor-led and self-directed formats. There is no license fee to offer the program, and no equipment is needed. Both the instructor-led and the self-directed formats focus on the WWE guidebook, which includes self-assessments, action plans, health education, motivational tools, and resources for maintaining an active lifestyle. Instructors receive online training and offer the program 3 times a week for 6 weeks.

The small grants program aimed to help organizations implement WWE successfully, document challenges and lessons learned during the 1-year installation period, and, ultimately, embed WWE in their organizations long term. The OAAA provided marketing and implementation resources at https://oaaction.unc.edu/resource-library/for-community-partners/, offered one-on-one support to grantees, and organized monthly group calls where grantees could discuss experiences, successful strategies, lessons learned, and plans for sustainability.

## Method

Our qualitative evaluation involved semistructured interviews with WWE program managers at grantee organizations to examine characteristics of the early implementation period. These CBOs represented diverse sectors ranging from health care to recreation and aging services. Interviews were conducted during the spring of 2018, approximately 8 months into the organizations’ grants.

### Theoretical Framework

Early implementation activities, like building organizational capacity and integrating a program with other activities, are vital steps toward program sustainability (Chamberlain et al., 2011; Conte et al., 2017; Fixsen et al., 2009). Since the OAAA’s goal was to help organizations embed WWE into their regular programming, we aimed to evaluate the early implementation period in the context of preparation for sustainability. Shediac-Rizkallah and Bone’s (1998) Planning for Sustainability framework was appropriate because it posits that the implementation process—alongside organizational characteristics and community context—affects program sustainability in potentially modifiable ways. The framework’s 11 factors for sustainability planning (Table 1) guided construction of our interview guide and served to organize themes that emerged during interviews.

**Table 1:** Interview Questions Mapped to Planning for Sustainability Framework Constructs

*Planning for Sustainability framework construct*	*Interview questions*
Program-level factors	
Project negotiation process and project effectiveness	What factors influenced your decision to apply for a WWE grant? Why did you choose WWE versus choosing to implement or expand another program? Now that you are in the process of implementing the WWE program, how have your beliefs about the program compared with your experiences in implementing it?
Project duration	Knowing what you know now, what do you see as the major issues to address when you have only a 1-year grant?
Project financing	How have the costs of implementing the WWE program been different from those you expected in your grant proposal?
Project type	We did not examine this construct, as all projects implemented the same program.
Training	How did your experience recruiting and retaining WWE leaders compare with your expectations when you received your grant? Knowing what you know now, what changes, if any, would you have made to your process of recruiting and retaining leaders?
Organization-level factors	
Institutional strength	What was the general receptivity to the WWE program by staff and leaders within your organization when you applied for the grant? Given your experiences with implementing WWE under this grant, how has the receptivity by staff and leaders changed since the grant began?
Integration with existing programs/services	How well have WWE activities fit with existing work processes and activities in your setting? What changes, if any, have you made within your organization during this grant period to integrate the WWE program with existing functions? What changes or alterations, if any, have you made to the WWE program format so that it would work well in your organization’s setting?
Program champion/leadership	In your application, you described which individuals in your organization would be completing various parts of the project. Did it turn out that way? Have any informal champions, people not employed at your organization, played a role in promoting the WWE program?
Community-level factors	
Socioeconomic and political considerations	What kinds of resources, if any, are available in your community that may help organizations like yours sustain the program long term? Have you accessed these resources already or do you plan to do so?
Community participation	To what extent do you network with other organizations that may be interested in the WWE program outside your setting?

*Note.* WWE = Walk With Ease.

### Participant Recruitment

We selected for the interview three grantees that exceeded 30% of their goal WWE enrollment at their 6-month progress report (henceforth “high-enrollment organizations” or “HEOs”) and two that had not enrolled any WWE participants at the same time point (henceforth “low-enrollment organizations” or “LEOs”; Table 2). By interviewing those with the most and the least enrollment success, we hoped to isolate key differences in organizational characteristics and the early implementation process that may have contributed to their varying success (Brinkerhoff, 2003).

**Table 2:** Selection of Interviewees Based on Participant Enrollment at 6 Months

*Grantee selected as HEO/LEO/not selected* ^a^	*% of goal participants reached*
HEO	64.00
HEO	31.41
HEO	30.50
Not selected	25.56
Not selected	23.45
Not selected	20.00
Not selected	18.67
Not selected	7.60
LEO	0.00
LEO	0.00

*Note.* HEO = high-enrollment organization; LEO = low-enrollment organization.

a All 10 grantees that began their Osteoarthritis Action Alliance grant period in April 2017 were considered for interviews based on enrollment numbers provided in 6-month progress reports.

We invited WWE program managers at the five target organizations to participate in telephone interviews. All organizations, though not all individuals, agreed to participate. Program managers were informed that the interview was voluntary and separate from OAAA grant activities and then asked to provide informed consent before participating. We conducted a single interview per organization, with one to four program managers participating. By interviewing all program managers simultaneously, we hoped to gain an organizational, rather than individual, perspective on the early implementation process. This study was deemed exempt as not human subjects research by the Institutional Review Board of the University of North Carolina at Chapel Hill, as the participants were interviewed from an organizational rather than an individual-level capacity.

### Interview Tool

Two senior-level PhD researchers and a research assistant from the OAAA developed a telephone interview guide with approximately 20 questions (varying, depending on answers). Questions assessed grantees’ experiences within the 11 constructs of the Planning for Sustainability framework, including organizational readiness to implement, perceptions about WWE’s effectiveness and compatibility with other organizational programming, and use of internal or external program champions or community partners during the early implementation process (Table 1).

### Interview Procedure

Telephone interviews were conducted by one trained interviewer, who was familiar with qualitative research procedures and with the OAAA small grants program but who had not been the grantees’ previous OAAA contact. Prior to the interview, program managers received an agenda of interview topics by email, though not the specific interview questions themselves. Interviews lasted for approximately 45 minutes, were recorded with permission, and transcribed.

### Data Analysis

We coded interview transcripts using NVivo 11 Data analysis software (QSR International, 2015). Coding proceeded by an integrated approach, employing a “start list” of codes reflecting theoretical constructs but allowing additional codes to emerge during the process (Bradley et al., 2007; Miles et al., 2013). One research assistant coded all transcripts, and two senior researchers independently reviewed and corroborated the transcripts, coding scheme, and findings. To analyze for common themes, we examined data by topic rather than examining individual organizations case by case. In addition, we grouped organizations’ responses by HEO/LEO status to examine differences in early implementation experiences for those with higher and lower enrollment numbers.

## Results

Only one of the three HEOs reached half its enrollment goal 6 months into its grant; the other two reached slightly over 30% (Table 2). The two LEOs did not enroll any participants in the first 6 months of their grants. We will describe common themes related to early implementation that emerged during interviews, highlighting where HEO and LEO organizations differed. We will then discuss barriers to implementation and provide real-world strategies that organizations employed to overcome them.

### Early Implementation Experiences: Common Themes

**Figure 1:**
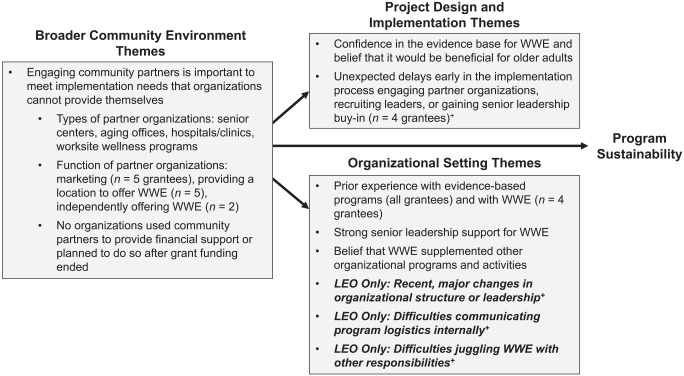
Common Themes* Related to Early Implementation for Walk With Ease (WWE), Organized According to the Planning for Sustainability Framework (Shediac-Rizkallah & Bone, 1998) *These themes were reported by all organizations unless otherwise noted. Themes that are bold and italicized were shared only by low-enrollments organizations (LEOs). ^+^These themes were also identified as barriers to WWE Implementation; see Table 3 for solutions that grantees used to overcome these barriers.

The organizations we interviewed shared many characteristics and WWE implementation strategies (Figure 1). Every organization had experience offering evidence-based programs for older adults. Program managers at every organization expressed confidence in WWE’s evidence base, thought that WWE was a beneficial supplement to their other programming, and stressed the importance of senior leadership support to facilitate WWE implementation. Four of five organizations had prior experience offering WWE—all the HEOs and one LEO. Program managers noted that having experience with WWE enhanced organizational and community buy-in (“the community had already embraced and was slightly aware of the program”), provided knowledge of how to implement the program (“we were able to dive into it because we already had prior experience”), and improved execution of plans for training leaders (“it was both the expectation and the reality that staff would be responsible for taking the training and facilitating their instructor-led classes”).

Additionally, every organization engaged community partners, though for different purposes. At all organizations, community partners offered locations for WWE groups to meet and provided marketing assistance. Two organizations also engaged community partners to independently offer the program. No organization relied on community partners for financial support or planned to do so after grant funding ended.

All but one organization reported unexpected delays during early implementation, due to difficulties in engaging senior leadership, recruiting WWE leaders, or recruiting partner organizations. One organization expressed a desire to lengthen the grant to allow time to manage unexpected difficulties:

We wish it was an 18-month grant. . . . We needed about four or five months to get everything up and going and to be able get everybody on board. Because you know as well as I do when you’re dealing with the powers that be, it’s a long process, but then once you get to the top, it was smooth sailing after that.

The organization that did not report delays was the same one that exceeded 50% of its goal participant reach. Program managers spoke of their existing experience with WWE as the means for success under this time line:

We had already had . . . about three years prior experience of working with the program, embedding it into our system. . . . I think it worked to our advantage that we already had Walk with Ease presence within the region.

### Early Implementation Experiences Unique to Low Enrollment Organizations

While common themes regarding early implementation emerged from all organizations, the two LEOs shared three organizational characteristics not reported by any HEOs:

1. *Substantial changes in organizational structure or leadership*: Both LEOs began their WWE grants in the midst of major organizational changes. One LEO experienced an organizational merger at the same time as the WWE grant, while the other reported significant senior leadership turnover at the beginning of the grant period.
2. *Difficulties in communicating logistics within their organization*: Technological and communication difficulties between different organizational departments delayed program implementation at both LEOs.
3. *Difficulties in balancing WWE and other program responsibilities*: At one LEO, a program manager admitted, “I do multiple programs, so I have to juggle, you know . . . so, yeah, I’ve had to put some things on the back burner.” At the other organization, a program manager reported, “I have been, you know, a little bit more lax than I have with other programs . . . not due to the curriculum it’s just due to my time constraint and . . . my priorities.”

### Barriers to Early Implementation and Solutions to Address Them

All organizations reported experiencing implementation barriers in the early months of their WWE grants, and none adhered exactly to their original implementation plans. Barriers reflected various factors, including geography, climate, organizational structure and staffing, and potential participants’ needs. However, organizations experienced many common barriers and often employed similar solutions to address them (Table 3). Three reported solutions were particularly relevant, as program managers used them to address more than one barrier:

1. *Adapt the instructor-led program to be offered 2, instead of 3, times per week*: Program managers from four organizations, unprompted by the interviewer, recommended that the instructor-led WWE program be offered twice instead of 3 times per week. Organizations recommended this adaptation in response to difficulties in engaging older adults and recruiting program leaders, as it would reduce strain on their time. One organization actually implemented the adaptation despite the reduction in program fidelity it caused:
  With some sites . . . sometimes they can’t commit to three days a week and that’s what the evidence shows is best, so we have encouraged them to start with two and then move up to three and that really has been helpful, and we can start recording the data when it gets to the three days a week.
2. *Utilize community partnerships*: Partnership with other CBOs was the most common way in which each organization addressed implementation barriers. Every grantee had initially planned to partner with organizations like senior centers, aging services, hospitals, clinics, and worksite wellness programs; however, the exact role of community partners changed as the specific needs became clearer. Community partners were particularly useful in solving resource barriers: They provided indoor walking space for winter classes, offered audiences for marketing WWE, and helped reach participants over large geographic areas.
3. *Train organizational staff as WWE leaders, instead of volunteers*: All grantees were required to train WWE instructors as part of their grant. Three organizations trained only their or partners’ staff, while two also relied on volunteers. Both organizations that trained volunteers experienced barriers, including difficulties in recruiting volunteers, lack of long-term commitment, and/or inability to provide incentives:
  They couldn’t offer up that voluntary work and we didn’t have the opportunity to reimburse them for things like travel or provide some incentive for that leader.
  Maybe I wasn’t expecting it to be as hard . . . I had a few people say that they were really interested in volunteering to be trained to teach the class, and then one person dropped out . . . two people were trained and one person halfway through dropped out. . . . I didn’t realize it would be that time consuming.
  [Program manager] trained three people and in the end only one person ended up teaching it, and then after the six-week session they were like I don’t want to teach it again.

**Table 3:** Barriers to WWE Implementation and Solutions Employed by Grantees

*Barriers to implementation*	*No. of CBOs reporting*	*Example quote*	*Solutions grantees used*
Large geographic area	2	“Three times a week to meet with individuals in the community and it’s a very geographically large area that we were instituting the project within.”	• Train WWE leaders at geographically distant partner organizations
Uncooperative (cold, hot, rainy, or snowy) weather	3	“Only one of our senior centers have an indoor walking path, which it’s hard to teach a walking program without . . . if it’s too hot or too cold or even if it’s not a safe walking environment outside.”	• Partner with organizations that have indoor walking locations
Difficulties in engaging partner organizations	2	“It took us a long time to get the right people in the room at the right time . . .that’s I think something that we learned or we’re reminded of is that, oh yeah, you can’t just make a community partner overnight.”	• Train interns to work with and provide support to partner organizations • Ensure early on that partnerships are mutually beneficial and that visions and goals align
Inability to provide incentives to WWE leaders	2	“We really were . . . challenged with providing incentives for the leaders. You know, to ensure their long-term participation.”	• No solutions reported this grant cycle, but grantees requested that the grant requirements be relaxed to allow for this
Difficulties in working with older adult populations	2	“Sometimes our older adults are a little bit more difficult to get walking, they need special encouragement.”	• Suggest that WWE could offered twice a week
Responsiveness/willingness of volunteer WWE leaders	2	“[Program manager] trained three people and in the end only one person ended up teaching it, and then after the six-week session they were like I don’t want to teach it again.”	• Train organizational staff • Cross-train dedicated volunteers leading other programs • Suggest that WWE be offered twice a week
Lengthy administrative approval processes	2	“Being a public entity it’s very hard to be able to cut through that red tape, so for about ten months we worked with our county administrator, the county council, our insurance carrier and finally have gotten an incentivized wellness program.”	• Slowed process at the outset, grantees were able to work through and begin recruitment; however, they suggested beginning administrative processes earlier
Big structural changes within the organization	2	“We are a huge . . . organization, plus we were right in the middle of a merger which did not help things at all.”	• No solutions reported
Other commitments draining staff time	2	“I have been a little bit more lax than I have with other programs . . . just due to my time constraint and you know where my priorities and where I need to get things done.”	• No solutions reported
Difficulties in communicating internally	2	“There was a lot of back and forth with our payment system and then the organization that did the training, so a little out of my hands.”	• Begin administrative processes earlier

*Note.* CBO = community-based organizations; WWE = Walk With Ease.

## Discussion

During the first 6 months of their WWE grants, every organization experienced unexpected barriers, and only one reached more than 50% of its recruitment goal. Many early implementation barriers mirrored those experienced by Oregon State University extension offices in 2012–2013 when they implemented WWE at county offices across Oregon (Conte et al., 2017). In that study, implementation was delayed by winter weather, insufficient recruitment and retention of volunteer leaders, and other commitments that pulled staff focus from WWE (Conte et al., 2017; Conte et al., 2019). Like the OAAA grantees, more than half of the new volunteer leaders in the Oregon study did not deliver any WWE programs, resulting in the recommendation to rely on either experienced volunteers or organizational staff (Conte et al., 2019).

Beyond the Oregon State University study, our findings mirror implementation experiences for other evidence-based programs for older adults, including Fit & Strong!, EnhanceFitness, and the Chronic Disease Self-Management Program (Belza et al., 2015; Derananian et al., 2012; Paone, 2014; Petrescu-Prahova et al., 2016). As in our study, barriers to implementing these programs included competition with other programs for physical space or staff time (Belza et al., 2015; Derananian et al., 2012; Petrescu-Prahova et al., 2016), difficulties in finding qualified instructors (Derananian et al., 2012), and difficulties in engaging older adults as participants (Derananian et al., 2012; Paone, 2014). Unlike our study, ongoing program funding was a common barrier in other programs—a topic OAAA grantees had yet to experience (Derananian et al., 2012; Paone, 2014; Petrescu-Prahova et al., 2016).

Organizations implementing other programs also used implementation strategies similar to those of the grantees in our study, including evaluating available resources, like physical space and staff time (Belza et al., 2015; Derananian et al., 2012; Paone, 2014), training and retaining strong program leaders (Belza et al., 2015; Paone, 2014; Petrescu-Prahova et al., 2016), utilizing community partners to improve participant reach (Belza et al., 2015; Paone, 2014), identifying internal program champions (Paone, 2014), and assessing potential program adaptations that would improve program-organization fit (Petrescu-Prahova et al., 2016). However, our study differs from studies of other evidence-based programs because it explicitly identifies differences in early implementation between organizations with high and low participant enrollment.

Most organizations in our study experienced implementation barriers that slowed WWE enrollment; however, these delays should not necessarily be considered wasted time. Rather, actions taken to surmount barriers may be useful as capacity-building activities. Implementation literature in health promotion variously define capacity building as (1) rendering an organization more able to address current and future health issues, (2) creating increased problem-solving capabilities (e.g., enhancing an organization’s community engagement network or health promotion expertise), or (3) improving organizational skills, motivations, knowledge, or attitudes toward implementing innovations (Flaspohler et al., 2008; Hawe et al., 1997; Labonte et al., 2002). From these definitions, it is clear that many barriers that WWE grantees experienced involved capacity-building activities—for example, difficulties in engaging senior leadership, recruiting WWE leaders, and recruiting partner organizations. The trial-and-error process that grantees employed to surmount these barriers, and the solutions that resulted from their experiments, built health promotion capacity for ongoing program implementation. Since capacity building is widely held as a critical component of intervention sustainability, organizations that provide grants to implement health promotion interventions should consider measuring capacity-building activities in addition to enrollment numbers as indicators of implementation success (Hawe et al., 1997; Labonte et al., 2002).

Our research also confirms the vital role of community partners in reaching participants and providing resources that organizations lack. Community partnerships sometimes took longer than expected to establish; however, their critical role in grantees’ implementation experiences suggests that partners offer benefits well worth the effort to bring them on board. It follows that organizations funding implementation efforts should emphasize partnerships and provide grantees sufficient time to establish them.

In this study, one especially relevant aspect of organizational capacity was existing experience with WWE. It is notable that one LEO with prior experience still failed to recruit any participants by the 6-month point in the grant. However, this grantee coordinated with a partner that lacked prior WWE experience and that suffered delays in gaining senior leadership support and surmounting administrative and communication hurdles.

Just as all HEOs had prior experience with WWE that facilitated implementation, the LEOs shared several barriers not reported by any HEOs. They each experienced recent, major structural changes and reported difficulties with internal communication, suggesting that implementing a new program may be complicated when existing processes are in flux or not functioning well. They also reported challenges in juggling WWE with other responsibilities. Just as prior experience with WWE enhanced capacity to implement WWE for the HEOs, these characteristics in the LEOs likely reduced organizational capacity to implement.

Our findings build on existing literature by offering concrete solutions to common early implementation barriers. Difficulties recruiting and retaining volunteer WWE leaders suggest that, where possible, organizations should use staff instead. Additionally, organizations’ frequent suggestion to adapt the instructor-led program to twice a week indicates the need for additional research to validate this adaptation or to explore other adaptations to improve program-organizational fit. The OAAA began this effort to improve program flexibility by working with the Arthritis Foundation and the CDC to determine acceptable program adaptations during this grant period. It was determined that an “enhanced self-directed” format could be offered whereby participants would read the guidebook on their own but congregate with other participants 1 to 3 times per week for group walks. In some cases, organizations provided a trained WWE leader to lead the walking activity and informally review guidebook content.

### Limitations

A few study limitations should be considered:

1. *Use of enrollment numbers as the sole measure of early implementation success*: We interviewed organizations with the highest and lowest WWE enrollment halfway into their grants, aiming to hear varying early implementation experiences. However, as this study and others indicate, enrollment is only one part of the early implementation experience and does not adequately measure capacity-building activities that are vital for program sustainability (Hawe et al., 1997). High and low enrollment should not be interpreted as the sole measure of implementation success.
2. *Evaluating only OAAA grantees limits generalizability*: Grantees were selected from a pool of applicants and were more likely than the general population of CBOs serving older adults to have experience with WWE or other evidence-based programs. Additionally, grantees received external support from the OAAA through monthly calls and participated in a learning collaborative with other grantees. Therefore, implementation actions, plans for sustainability, and solutions to barriers may over-reflect strategies endorsed by the OAAA.
3. *Small sample size may allow for random bias*: This study relied on interviews with only five OAAA grantees. However, we maximized the variety of our sample by selecting grantees with the highest and lowest enrollment by the date of their 6-month progress report (Brinkerhoff, 2003).

### Conclusion and Implications

Understanding how to successfully implement arthritis-appropriate, evidence-based programs in CBOs, and to sustain them after initial funding ends, is important for grant funders, organizational leaders investing time and effort in programs, and community members with arthritis who can benefit from them. This investigation examined early implementation experiences for CBOs that experienced the highest and lowest enrollment among OAAA small grant recipients. The results of this study could inform organizations in developing plans to implement WWE, anticipating barriers, and strategizing solutions to overcome them. Given similarities between implementation experiences in this study and studies of other evidence-based programs, our study could also inform implementation research for other programs—particularly regarding addressing implementation barriers. Our results also suggest avenues for future research in WWE—particularly the need to test adaptations that could improve the program’s flexibility and enhance the likelihood of WWE’s long-term uptake within organizations.
